# RNA dysfunction in age-related macular degeneration: the role of U1 snRNP complex and neurodegenerative diseases

**DOI:** 10.1186/s40942-025-00736-8

**Published:** 2025-10-16

**Authors:** Juliana M. Bottos, Ericks S. Soares, Camila G. M. Zimmer, Vanessa V. C. Sinatti, Caio B. Q. S. Leal, Juliana M. F. Sallum

**Affiliations:** 1 Department of Ophthalmology, Federal University of São Paulo (UNIFESP), São Paulo, Brazil; 2 Aptah Bio Inc, MBC BioLabs, 930 Brittan Avenue, San Carlos, 94070 USA

**Keywords:** Age-related macular degeneration, RNA splicing, U1 snRNP, Spliceosome, Neurodegenerative diseases, Aging, Cotranscription, Long genes

## Abstract

**Background:**

Age-related macular degeneration (AMD), a leading cause of vision loss in elderly individuals, is a multifactorial disease driven by genetic, environmental, and cellular aging processes. Emerging evidence highlights the critical role of ribonucleic acid (RNA) splicing dysfunction in AMD pathogenesis, with a focus on the U1 small nuclear ribonucleoprotein (U1 snRNP) complex, a key spliceosome component. U1 snRNPs ensure the fidelity of RNA cotranscription and pre-mRNA splicing initiation, and their dysfunction has been implicated in neurodegenerative disorders and other age-related diseases.

**Main body:**

This narrative review explores the impact of U1 snRNP dysregulation on retinal cells, focusing on its role in transcriptomic instability, impaired protein homeostasis, cellular stress, impaired autophagy, and inflammation, which are important features of AMD pathogenesis. Finally, we propose that targeting U1 snRNP dysfunction could provide a novel therapeutic approach to slow, prevent, or restore retinal degeneration, offering insights into broader implications for age-related diseases.

**Short conclusion:**

Understanding the molecular mechanisms underlying U1 snRNP dynamics in retinal health and degeneration is essential for developing innovative and effective treatments for AMD, which may provide ways to delay or reverse the effects of aging and associated diseases.

## Background

Age-related macular degeneration (AMD) is a progressive neurodegenerative disease influenced by multiple factors, including aging, genetics, and environmental conditions [1]. It is the leading cause of irreversible central vision loss in elderly individuals, and its prevalence is expected to increase significantly owing to the aging population and increasing life expectancy. In 2020, 196 million people were affected by AMD worldwide, and this number is projected to reach 288 million by 2040 [2]. AMD manifests in two primary forms: dry (nonexudative) and wet (exudative) AMD. The dry form, accounting for approximately 90% of all cases, is characterized by the gradual accumulation of drusen beneath the retina, leading to atrophy of the retinal pigment epithelium (RPE) and loss of photoreceptors (PR), which results in a slow, progressive decline in central vision. Conversely, wet AMD, although less common, is more severe and characterized by abnormal growth of choroidal blood vessels (choroidal neovascularization) beneath the retina. These fragile vessels leak blood and fluid, causing rapid retinal damage, scarring, and substantial vision loss [3, 4].

The treatment options for AMD vary by form. Wet AMD is often managed with intravitreal injections of vascular endothelial growth factor (VEGF) inhibitors to suppress abnormal blood vessel growth, or in combination with angiopoietin-2, to enhance vascular stability [5]. However, treatment for dry AMD is limited to strategies that slow the progression of AMD through lifestyle modifications, intravitreal complement modulation, and vitamin supplementation [6–9]. The primary therapeutic strategies for dry AMD that are currently under investigation include the following:

- complement pathway inhibitors
- drugs that target oxidative stress
- beta-amyloid antibodies
- neuroprotective small molecules
- visual cycle modulators
- stem cell therapies
- and anti-inflammatory agents [10]

However, the development of these treatments faces significant challenges, including an incomplete understanding of AMD pathogenesis, the complexity of delivering drugs to the retina, limited preclinical models, and the need for innovative clinical trial approaches and novel endpoints [6–10].

Addressing the complex pathophysiology of AMD will likely require multitargeted approaches rather than focusing on a single aspect. The increasing understanding of RNA mechanisms and their role in neurodegenerative diseases has spurred the development of biomarkers and innovative therapeutic strategies [11].

This article is a narrative, integrative review that synthesizes current evidence from molecular biology, retinal degeneration, and neuroscience to construct a novel theoretical framework regarding the role of RNA splicing dysfunction in AMD, with a particular focus on the U1 snRNP complex. Relevant studies were identified through a comprehensive search of peer-reviewed literature using databases such as PubMed and Web of Science. Keywords included “age-related macular degeneration,” “RNA splicing,” “U1 snRNP,” “spliceosome,” “neurodegenerative diseases,” “aging,” “cotranscription,” and “long genes.” Articles were selected based on their conceptual relevance, methodological rigor, and contribution to understanding molecular mechanisms of neurodegeneration. This synthesis aims to construct a cohesive pathogenic framework that supports future therapeutic strategies targeting the dysfunction of the U1 snRNP complex in AMD and related disorders.

## The RNA biology

When Sydney Brenner first reported the discovery of messenger RNA (mRNA) in 1961, it was considered merely a molecule that acted as a bridge between deoxyribonucleic acid (DNA) and proteins to transfer genetic information [12]. Currently, at least 15 distinct types of RNA molecules reveal unique features of the RNA landscape [13].

The human transcriptome is the complete set of RNA molecules transcribed from the human genome. It represents all the RNA content in a particular cell, tissue, or organism at a specific time, reflecting the genes actively expressed under certain conditions.

The transcriptome can generally be separated into two categories: coding RNA, which is represented by mRNAs and accounts for 4% of the total RNA, and noncoding RNA (ncRNA), which accounts for the remaining 96% [14]. ncRNAs are further separated into housekeeping ncRNAs and regulatory ncRNAs. Housekeeping ncRNAs include transfer RNAs (tRNAs), ribosomal RNAs (rRNAs), small nucleolar RNAs (snoRNAs), and small nuclear RNAs (snRNAs) [15]. Regulatory ncRNAs include small noncoding RNAs (fewer than 200 nucleotides in length) and long noncoding RNAs (more than 200 nucleotides) [16]. Table 1 presents an overview of the categories of coding and ncRNAs and their primary functions.

**Table 1:** Primary categories of coding and noncoding RNAs

RNA Class	Description	Molecular Function
*CODING RNA*		
*mRNA*	Messenger RNA	Template for protein synthesis
*NON-CODING RNA*		
*Housekeeping ncRNA*		
*tRNA*	Transfer RNA	Delivers amino acids to the ribosome for protein assembly
*rRNA*	Ribosomal RNA	Component of ribosomal subunits, catalyzes peptide bond formation
*snoRNA*	Small nucleolar RNA	Guides chemical modifications of rRNA, tRNA, and snRNA
*snRNA*	Small nuclear RNA	Component of the spliceosome, catalyzes pre-mRNA splicing
*Regulatory ncRNA*		
*Long noncoding RNA ( > 200 nt)*		
*ceRNA*	Competing endogenous RNA	Regulates gene expression by competing for miRNA binding
*circRNA*	Circular RNA	miRNA decoys, transcription regulators, interference with splicing
*lincRNA*	Long intergenic noncoding RNA	DNA–chromatin complex scaffolds
*NATs/OS*	Natural antisense transcripts/opposite strand	Transcriptional regulation in cis or trans
*Small noncoding RNA ( < 200 nt)*		
*miRNA*	microRNA	Posttranscriptional silencing, translational repression
*piRNA*	PIWI-interacting RNA	Silences transposons, regulates epigenetic modifications in germline cells

Abbreviations: *mRNA: messenger RNA, ncRNA: noncoding RNA, tRNA: transfer RNA, rRNA: ribosomal RNA, snoRNA: small nucleolar RNA, snRNA: small nuclear RNA, ceRNA: competing endogenous RNA, circRNA: circular RNA, lincRNA: long intergenic noncoding RNA, NATs/OS: natural antisense transcripts/opposite strand RNA, miRNA: microRNA, piRNA: PIWI-interacting RNA*

### RNA splicing

**Fig. 1:**
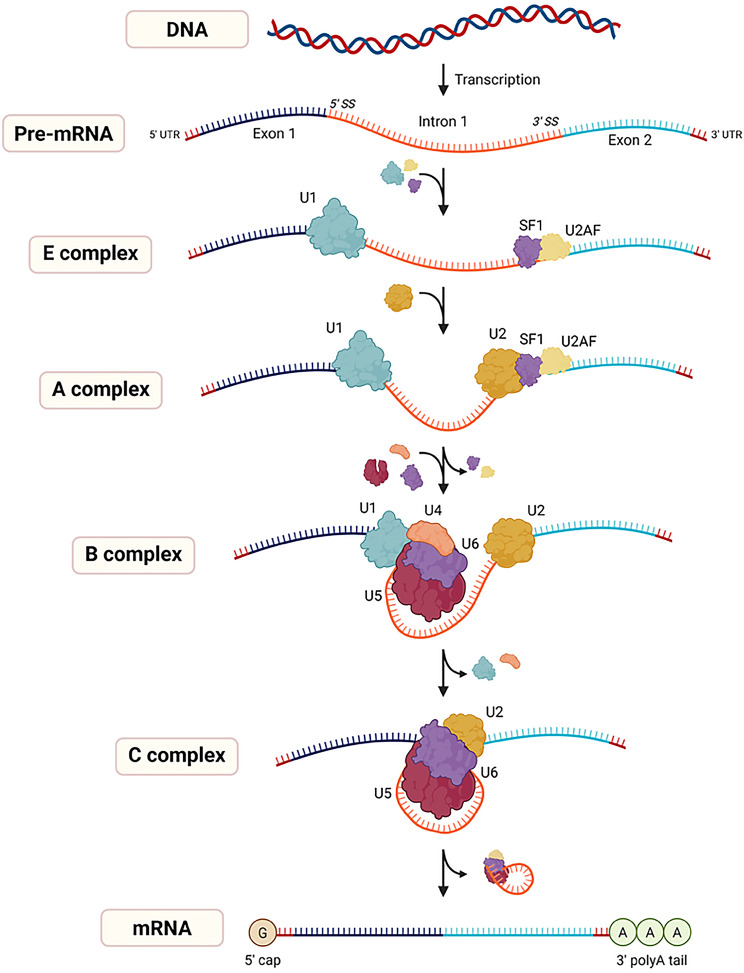
RNA splicing is a tightly regulated process in which the spliceosome, a dynamic snRNPs complex (U1, U2, U4, U5, and U6) and associated proteins, recognizes specific splice sites in pre-mRNAs, to remove introns and join exons, producing mature mRNAs. This mechanism ensures accurate and flexible RNA processing, contributing to transcriptome diversity. UTR: untranslated region. *created with*BioRender.com

RNA splicing was first described by Richard J. Roberts and Phillip A. Sharp in 1977 [17–20]. They independently discovered that, in contrast to simpler bacterial genes, complex genes in eukaryotic cells are divided into segments known as coding regions (exons) and noncoding regions (introns). During transcription, both gene regions are transcribed into precursor mRNAs (pre-mRNAs). RNA splicing involves the removal of introns and the joining of exons from pre-mRNAs to create mature mRNAs. The mRNA is then exported to the cytoplasm and translated into a protein [21] (Fig. 1). The discovery of this process has transformed the understanding of gene expression and structure. This groundbreaking discovery earned Roberts and Sharp the 1993 Nobel Prize in Physiology or Medicine [17, 20].

### The spliceosomal machinery

Splicing is accomplished by a large macromolecular complex (~3 MDa) known as the spliceosome. This intricate macromolecular machine comprises five snRNPs—U1, U2, U4, U5, and U6—and numerous proteins with a dynamic structure and composition [22, 23]. Over 170 proteins are associated with the core splicing machinery at various stages of the splicing process [23], with each step being precisely regulated to support cellular homeostasis and maintain cellular fitness [24]. These processes are accompanied by extensive remodeling of the snRNPs within the spliceosome, conferring accuracy and adaptability to the splicing machinery [22].

The spliceosome acts as a molecular scissor, removing intronic regions from pre-mRNAs. It recognizes the intron‒exon boundaries of genes, which are defined by the 5′ splice donor, 3′ splice acceptor, and branch sites [25]. Spliceosome activity can be regulated by multiple splicing activators and repressor proteins, called regulatory splicing factors (SFs), which bind to enhancer and silencer elements in pre-mRNAs [26].

### U1 snRNP complex

The U1 snRNP complex is a key component of the spliceosome and is the most abundant ribonucleoprotein (RNP) complex in human cells. Each cell is thought to contain approximately one million copies of this complex [27], which is formed by a U1 small nuclear RNA (snRNA), seven Sm proteins, and U1-A, U1-C, and U1-70K snRNPs, which work together as functional units in pre-mRNA splicing [28].

The U1 snRNP complex is required for pre-mRNA splicing initiation and gene regulation, participating in 5′ splice site recognition, spliceosome assembly, splicing fidelity, alternative splicing (AS) modulation, and 3′ untranslated region (UTR) processing through cotranscriptional mechanisms (detailed in subsequent sections) [27]. In addition to its role in splicing, U1 snRNP interacts with RNA polymerase II (RNA Pol II), coordinating transcription dynamics and mRNA stability [29]. This functional coupling promotes efficient cotranscriptional splicing, prevents exon misprocessing, and contributes to the accuracy of gene expression. U1 snRNPs integrate splicing and cotranscriptional regulation to preserve transcriptome integrity, with a prominent function in maintaining the expression of long genes [29].

**Fig. 2:**
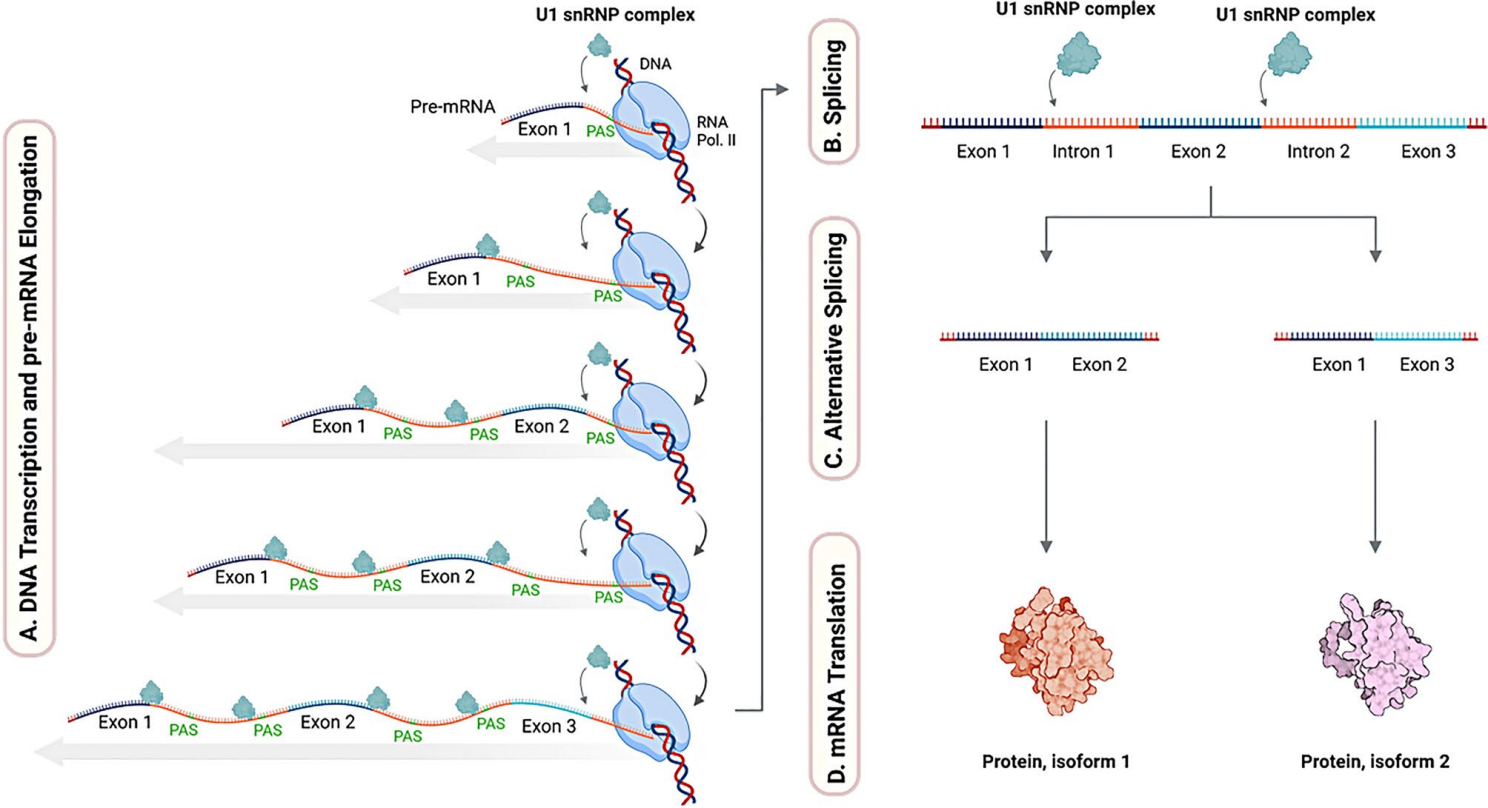
This figure illustrates the dual functions of U1 snRNPs in cotranscription and RNA splicing. This coupling is essential for gene expression and stability, especially in long genes. **A**. DNA transcription and pre-mRNA elongation—U1 snRnps interact with RNA polymerase II, coordinating transcription and mRNA stability, promoting cotranscriptional splicing, and preventing exon misprocessing. **B**. Splicing—U1 snRNP also binds to the 5′ splice site of pre-mRNA to initiate spliceosome assembly, ensuring the accuracy of splicing. **C**. Alternative splicing—a regulated process in which the spliceosome selectively includes or excludes specific exons, generating multiple mRNA isoforms from the same pre-mRNA. **D**. mRNA translation—the mechanism of alternative splicing increases proteomic diversity, allowing cell-specific protein expression and functional specialization by the generation of different protein isoforms. *created with* BioRender.com

U1 snRNP is the first small nuclear RNP to bind pre-mRNA, recognizing the 5′ splice site, which marks the exon–intron boundary, to initiate spliceosome assembly. This interaction guides the recruitment of additional snRNPs, including U2, and stabilizes the early spliceosomal complex, ensuring accurate splice site selection and efficient pre-mRNA splicing [30, 31] (Fig. 2).

Dysfunction of the U1 snRNP complex disrupts these tightly regulated processes, leading to widespread gene expression abnormalities. In splicing, its impairment results in intron retention, exon skipping, and the activation of incorrect or cryptic splice sites, generating aberrant mRNA isoforms. These defective transcripts can produce nonfunctional, truncated, or toxic proteins associated with genetic disorders, cancer, and neurodegenerative diseases [32]. Additionally, U1 snRNP dysfunction affects its interaction with RNA Pol II, leading to premature transcription termination, defective mRNA maturation, and instability, particularly in long genes [29]. This dysregulation not only alters protein diversity but also contributes to cellular stress, impaired homeostasis, and disease progression [32, 33].

### Alternative splicing

AS is a mechanism that generates multiple mRNA isoforms from a single pre-mRNA, thereby increasing proteome diversity. [34] This process contrasts with constitutive (regular) splicing, a default mechanism in which all introns are removed, and exons are joined together in a fixed, sequential manner to produce a single, specific mRNA transcript. This transcript encodes one specific protein product, as the same combination of exons is consistently retained [35].

In contrast, AS is a tightly regulated process in which the spliceosome selectively includes or excludes specific exons, creating multiple mRNA isoforms from the same pre-mRNA. This mechanism enables the generation of diverse protein isoforms, thereby expanding the proteome to produce cell-specific protein combinations that define the functional properties of different cell types [34].

The generation of unique mRNA isoforms through AS, coupled with the cotranscriptional function of U1 snRNPs to ensure stability, mainly in long genes, enables higher eukaryotes to achieve proteomic complexity without a proportional increase in gene number, highlighting the evolutionary importance of U1 snRNPs across species [36–39].

Although all cells require the function of the spliceosome, neural brain and retina cells are remarkably vulnerable to splicing perturbations owing to their complex cellular functions, which require specific splice isoforms. AS is relevant in specialized cells, where it supports neurogenesis, migration, and synaptic function [26, 34, 40, 41]. While AS enhances genetic plasticity, it also increases the risk of splicing errors, leading to functional disruptions [42]. As a result, numerous neurodegenerative diseases are linked to splicing defects [21, 43, 44].

Transcriptome analyses have revealed unprecedented levels of AS in retinal PR cells, suggesting a link between AS and light perception [11, 45–47]. The unique noncoding transcripts and isoforms in these cells highlight AS as a key factor in the transcriptional complexity of retinal gene expression, making the retina an ideal model for RNA biology research [11, 47–51].

### U1 snRNP cotranscription and premature polyadenylation

Polyadenylation (PA) is a two-step process that involves the cleavage of pre-mRNAs, usually at the 3’ UTR, and the addition of a polyadenosine (polyA) tail, which is fundamental for mRNA stability, nuclear export, and efficient translation. Premature PAs, where the polyA tail is added before complete transcript processing, can lead to the formation of truncated mRNAs and nonfunctional or harmful proteins [52].

The U1 snRNP complex suppresses premature cleavage and PA of pre-mRNAs by masking cryptic PA sites, ensuring transcript integrity [31, 32]. This U1 snRNP cotranscriptional process is essential for the full-length transcription of genes, particularly those with long introns, which are more susceptible to premature PA [27, 53, 54]. Both alternative splicing and U1 cotranscriptional processes are considered key drivers of evolutionary complexity [55], as they significantly impact the cellular regulatory landscape, protein diversity, and organismal complexity [56].

**Fig. 3:**
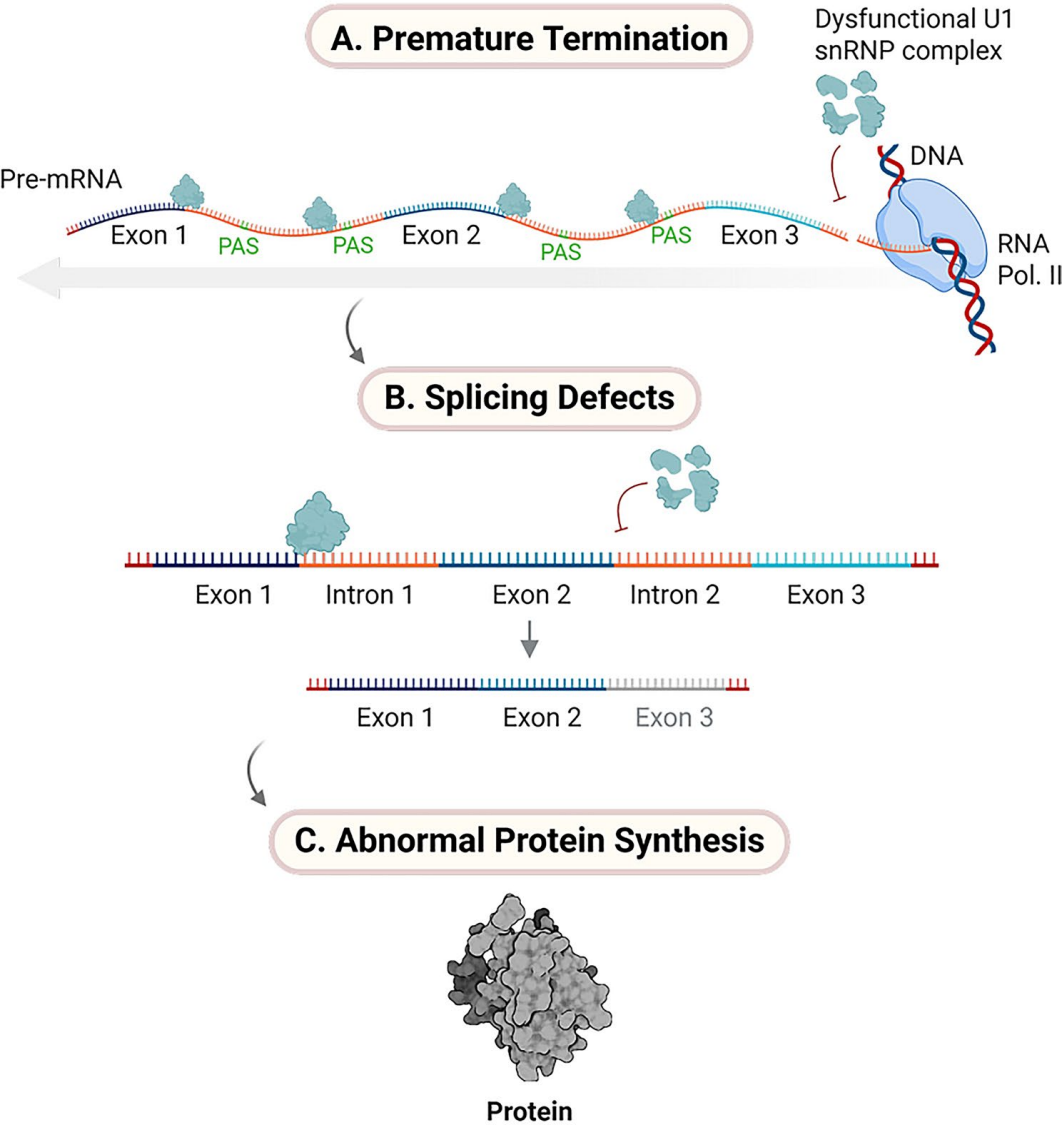
**A**. Premature termination. disruption of the cotranscriptional function of U1 snRNPs impairs their ability to suppress premature polyadenylation, leading to early cleavage of pre-mRNA transcripts. **B**. Splicing defects. this results in truncated, nonfunctional, or harmful mRNAs, particularly long genes with multiple splice variants. **C**. Abnormal protein synthesis. the loss of transcript integrity contributes to dysregulated protein expression and is implicated in various diseases. *created with* BioRender.com

Longer genes generate more splice variants with distinct functions [57], which require regulatory mechanisms to prevent premature cleavage. U1 snRNP protects these transcripts, ensuring full-length mRNA production. Loss of this protection can lead to dysfunctional, shortened transcripts [58]. This phenomenon is observed in various diseases. A moderate reduction in U1 snRNP levels leads to shorter transcripts, a pattern observed in stem cells and activated immune cells [59]. While this process supports normal cellular function, it may also increase protein production, which can activate oncogenes in cancer cells [52, 60]. In contrast, during development and differentiation, especially in specialized cells such as neurons in the brain and retina, longer mRNA transcripts are produced [52, 60–62]. The vulnerability of neurons to disturbances in U1 snRNP homeostasis may explain the prevalence of these defects in neurodegenerative disorders [24](Fig. 3).

### Epitranscriptome

Epigenetics refers to heritable changes in gene expression that do not involve alterations in the DNA sequence and are often mediated by mechanisms such as DNA methylation, histone modification, and chromatin remodeling [63]. In parallel, the term epitranscriptome refers to chemical modifications of RNA that regulate its metabolism without changing the RNA nucleotide sequence [64]. To date, over 150 different epitranscriptome modifications have been described for RNA, positioning the epitranscriptome as a key regulator of the transcriptional landscape, given that these modifications can disrupt RNA stability, splicing, and translation [11, 64–68].

Retinal cells, particularly RPE and PR cells, are highly susceptible to these epitranscriptomic changes, especially the N6-methyladenosine (m6A) modification [69]. As a neural tissue that is directly exposed to sunlight throughout life, with no turnover, high metabolic demand, and limited regenerative capacity, the retina faces cumulative damage from photooxidative stress. These chronic injuries increase susceptibility to RNA dysregulation, which can contribute to dry AMD by impairing cellular stress responses, immune regulation, cytokine expression, lipid metabolism, and complement system activation [70–73]. Numerous physical, chemical, and biological factors can induce oxidative stress, resulting in the generation of reactive oxygen species (ROS). Under normal conditions, ROS can act as effectors and signaling molecules; however, chronically, when produced in excess or mislocalized, they can impact the epitranscriptome [16]. In this way, oxidative stress particularly affects noncoding RNAs, causing abnormalities in their expression, which may contribute to the pathophysiology of many diseases, including cancer and neurodegenerative diseases such as Alzheimer’s disease (AD), Parkinson’s disease (PD), and AMD [16, 74–80].

Other environmental factors, such as nutrition, exercise, pollution, and smoking, also trigger the activation of specific cellular programs that respond quickly through changes in gene expression [47, 73, 81].

## RNA dysfunction in the developing and aging retina

Aging is the primary risk factor for major human pathologies. As a result, extensive research has focused on understanding the molecular basis of biological aging [82, 83]. Indeed, aging and many age-related diseases share similar hallmarks of RNA dysfunction, as AS alterations can occur in both healthy aging and several diseases [36, 84]. RNA processing and splicing are among the major categories of age-related differentially spliced transcripts shared across human tissues [84, 85].

**Fig. 4:**
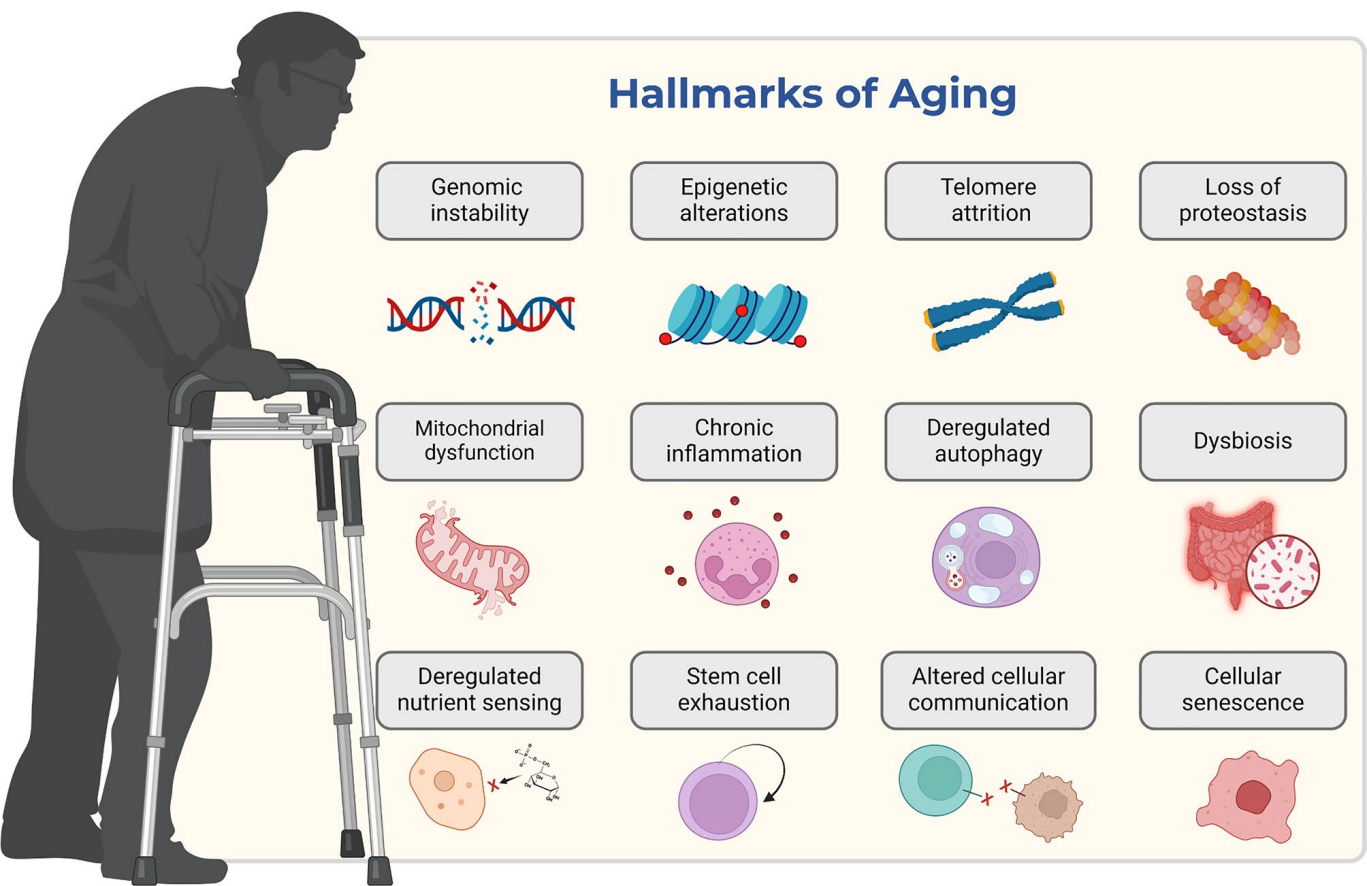
The key biological mechanisms of aging are related to the interconnected processes and molecular basis of biological aging that contribute to tissue decline and aging-related health deterioration. *created with* BioRender.com

During their lifetime, aging cells accumulate DNA mutations and unrepaired damage. Nonetheless, aging is not caused by a single type of damage. Despite differences between tissues, aging is associated with several hallmark modifications at the cellular and molecular levels, as shown in Fig. 4 [36, 83, 86–89]. These include some alterations as follows:

- epigenetic and epitranscriptome modifications
- changes in chromatin structure
- genomic instability
- telomere attrition
- metabolic changes
- lipid peroxidation
- misfolded proteins
- decline in proteasomal function
- impaired autophagy and phagocytosis
- accumulation of senescent cells
- chronic inflammation
- impaired cellular communication
- loss of stem cell renewal capabilities

These factors contribute to physiological decline, chronic diseases, and increased mortality. The brain and retina are highly susceptible to these injuries, but several repair mechanisms work to correct these events before they lead to mutations. However, the efficiency of these repair pathways decreases with age [82, 90–93].

There is a clear relationship between aging and altered U1 snRNP homeostasis [24]. With age, reduced U1 snRNP levels impair cotranscriptional processing, leading to premature transcriptional termination of long genes. This, in turn, results in the accumulation of nonfunctional RNAs, truncated proteins, and altered gene isoforms that compromise cellular function [36, 54, 59, 94], all of which share common hallmarks with neurodegenerative and age-related diseases [84].

The differential regulation of short and long genes with age appears to be a relevant factor in the aging process, affecting tissue health and potentially influencing the onset of age-related diseases [95]. During aging, the transcriptional machinery becomes less efficient and more prone to errors [36, 96, 97]. This inefficiency disproportionately affects long genes because their transcription requires more time and energy, increasing their vulnerability to interruptions. Short genes, otherwise, tend to be transcribed more quickly and may be less affected by these age-related inefficiencies, allowing them to maintain higher expression levels in aging cells than long genes [96]. Thus, shorter genes tend to either maintain or even increase their expression with age, whereas longer genes often show decreased expression [98]. Another mechanism contributing to the age-related decline in long-gene expression is chromatin remodeling that accompanies aging, which alters histone methylation patterns and reduces histone abundance, thereby changing chromatin compaction and accessibility [99–102]. Because long genes require coordinated regulation and a stable chromatin landscape to sustain transcriptional elongation, these changes promote polymerase pausing and premature termination, ultimately lowering their expression [95, 103].

Understanding these mechanisms is essential for developing strategies to mitigate age-related decline. One potential approach could involve discovering ways to support the transcription of critical long genes in aging cells, which may help preserve cellular function and delay the onset of age-related diseases, especially in tissues that depend on these genes for specialized functions, such as the brain and retina [95, 98, 104].

### Potential mechanism of AMD pathogenesis related to U1 snRNP dysfunction

The retina is a highly metabolically active tissue that is acutely susceptible to oxidative stress because of its continuous exposure to high levels of light and oxygen. Over time, aging and environmental agents exert a chronic, cumulative burden on RPE cells. These postmitotic cells are particularly vulnerable to damage, as they cannot dilute toxic byproducts through cell division. Age-related changes in the RPE include alterations in pigmentation, increases in lipofuscin granules, decreases in mitochondrial function, accumulation of proinflammatory substances, and decreases in RPE cell density due to apoptosis [105–109]. Oxidative stress in the RPE is also attributed to lipofuscin, which is a pigment granule composed of lipid-containing residues from lysosomal digestion that generate ROS upon blue light excitation [110]. ROS levels are controlled and maintained by the antioxidant system. However, when ROS levels surpass the antioxidant capacity of the cell, oxidative stress ensues [108, 111]. As a result of ROS overproduction and subsequent mitochondrial DNA damage, several mitochondrial proteins involved in the apoptosis cascade, such as cytochrome c and apoptosis-inducing factor, are released [112].

Oxidative stress and mitochondrial injury activate the intrinsic apoptotic pathway, culminating in cytochrome c release, apoptosome assembly, and the activation of caspase 9 and caspase 3. The extrinsic apoptotic pathway, triggered by inflammatory cytokines such as TNF-α, also converges on caspase 3 activation [108].

**Fig. 5:**
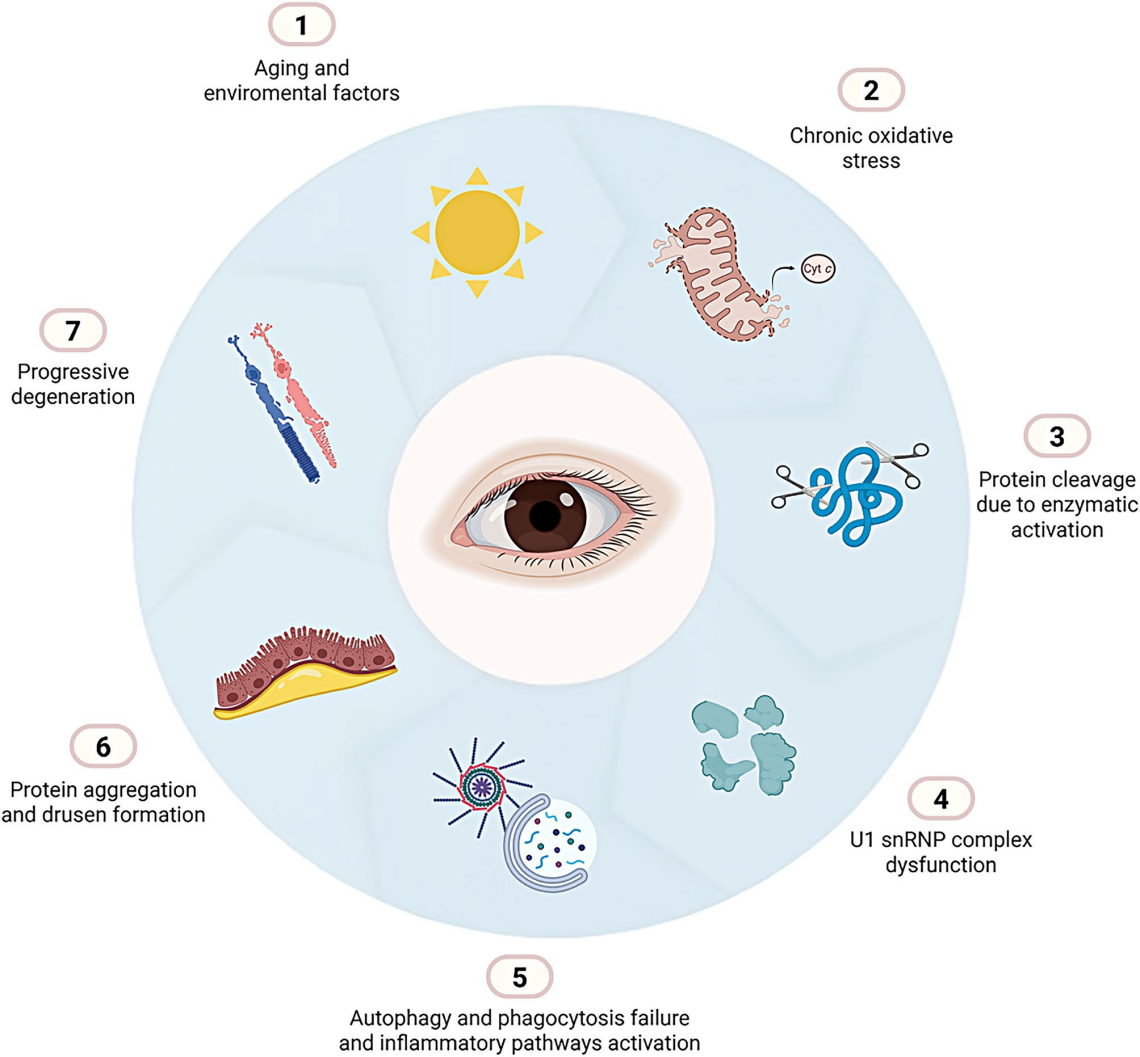
Pathogenic cascade in dry AMD associated with U1 snRNP dysfunction. (1) aging and environmental stressors chronically damage RPE cells. (2) persistent oxidative stress and mitochondrial injury induce cytochrome c release, apoptosome formation, and activation of caspases-3/9. (3) caspase-3 cleavage disrupts U1-70K and Sm proteins essential for U1 snRNP assembly, and targets beclin-1, ATG5, and MerTK, compromising autophagy and phagocytosis. (4) U1 snRNP dysfunction leads to defective splicing and transcript misprocessing. (5) impaired clearance mechanisms exacerbate debris accumulation and chronic inflammation. (6) misprocessed transcripts and protein aggregation promote drusen formation. (7) these processes reinforce a self-perpetuating cycle of cellular dysfunction that drives progressive retinal degeneration. *created with* BioRender.com

Activated caspase 3 cleaves essential nuclear proteins, including the U1–70k and Sm proteins, which are required for the proper assembly and function of the U1 snRNP complex [113]. These modified U1 snRNP components relocate to apoptotic bodies near the cell surface [114, 115]. These alterations may contribute to the breakdown of the mRNA splicing machinery during apoptosis and potentially trigger autoimmune responses in susceptible individuals [114]. Disruption of this complex impairs RNA splicing, leading to the accumulation of misprocessed transcripts, protein aggregation, and cellular stress. In the cytoplasm, caspase-3 also cleaves key regulators of autophagy and phagocytosis, including Beclin-1 and ATG5, which are essential for autophagosome formation, and MerTK, a tyrosine kinase crucial for the daily phagocytosis of PR outer segments by the RPE [116, 117]. Efficient autophagy is essential for maintaining homeostasis in RPE cells, as it allows for the clearance of damaged proteins and organelles [77, 78, 80, 118–121]. The cleavage of these proteins contributes to autophagy failure, phagocytic impairment, and the accumulation of cellular debris, further exacerbating inflammation and cell dysfunction [77, 80, 119–123]. Moreover, aging (the time of continuous chronic injury) and genetic variants in the complement system can exacerbate the progression of AMD, leading to earlier onset or increased severity (Fig. 5) [56, 108, 124, 125].

These interrelated processes establish a vicious cycle and support a model in which U1 snRNP dysfunction, triggered by oxidative stress and apoptotic cleavage, acts as a central driver of splicing failure, autophagy impairment, and neurodegeneration in dry AMD, highlighting these pathways as promising therapeutic targets.

### RNA spliceosome dysfunction in inherited retinal diseases

Inherited retinal diseases (IRDs) have placed the retina at the forefront of gene and RNA therapeutics owing to its surgical accessibility, relative immune privilege, and the ability to noninvasively track disease progression and treatment response [126]. While most IRDs are caused by single-gene mutations that primarily affect PRs and, less often, the RPE, an important subset arises from errors in pre-mRNA processing. Variants in spliceosome proteins—or in factors that regulate them—disrupt normal isoform production and impair retinal cell function. Because vision relies on the accurate splicing of long, highly expressed retinal transcripts, even small splicing defects tend to accumulate over time, driving progressive dysfunction and degeneration. These mechanistic insights have motivated RNA-directed interventions—allele-specific silencing with small-interfering RNA (siRNA), short hairpin RNA (shRNA), antisense oligonucleotides (ASO) to correct or modulate splicing, and engineered RNA-guided strategies (ERGS)—that can address variants previously considered untreatable [127].

Neural tissues are known to exhibit the greatest number of AS events [56], as variations in transcript isoforms, AS, and ncRNAs increase gene and phenotypic diversity and complexity, allowing cells to function distinctly from one another [47, 51]. The importance of AS in the retina has been demonstrated by numerous examples. In PR, AS is important for generating the protein diversity necessary for light detection, signal transduction, and cellular maintenance. Many PR-specific genes, including those encoding opsins (light-sensitive proteins) and components of the phototransduction pathway, undergo AS. Proper splicing is required for the precise function and structure of these proteins, which are critical for normal vision [124, 128].

Some SFs are specifically important for processing retinal transcripts since their mutations cause retinal dystrophy. Notably, most of these factors, including PRPF3, PRPF4, PRPF6, PRPF8, and PRPF31, are necessary for mediating interactions between U4/U6 and U5 snRNPs, which are fundamental components of the spliceosome machinery [56].

In addition to the previously mentioned genes, mutations in genes encoding SFs include SNRNP200, DHX38, PAP1, RPGR, BBS8, DYNC2H1, CEP290, CWC27, and others [129, 130]. All these genes are associated with a variety of IRD [131, 132], such as cone‒rod dystrophy, recessive Usher syndrome type 2, X-linked and dominant retinitis pigmentosa (RP), recessive Bardet–Biedl syndrome, recessive Senior–Loken syndrome, recessive Joubert syndrome, recessive Leber congenital amaurosis, recessive Meckel syndrome, syndromic retinal degeneration, and Stargardt disease [56].

Mutations in SFs can result in a phenotype restricted to the retina and other neural cells while being tolerated by other tissues. This phenomenon may be explained by the fact that the retina presents relatively high levels of certain specific and unique SFs that regulate PR-specific genes involved in phototransduction and the visual cycle [21, 56, 133, 134].

## U1 snRNP dysfunction in neurodegenerative and other diseases

**Fig. 6:**
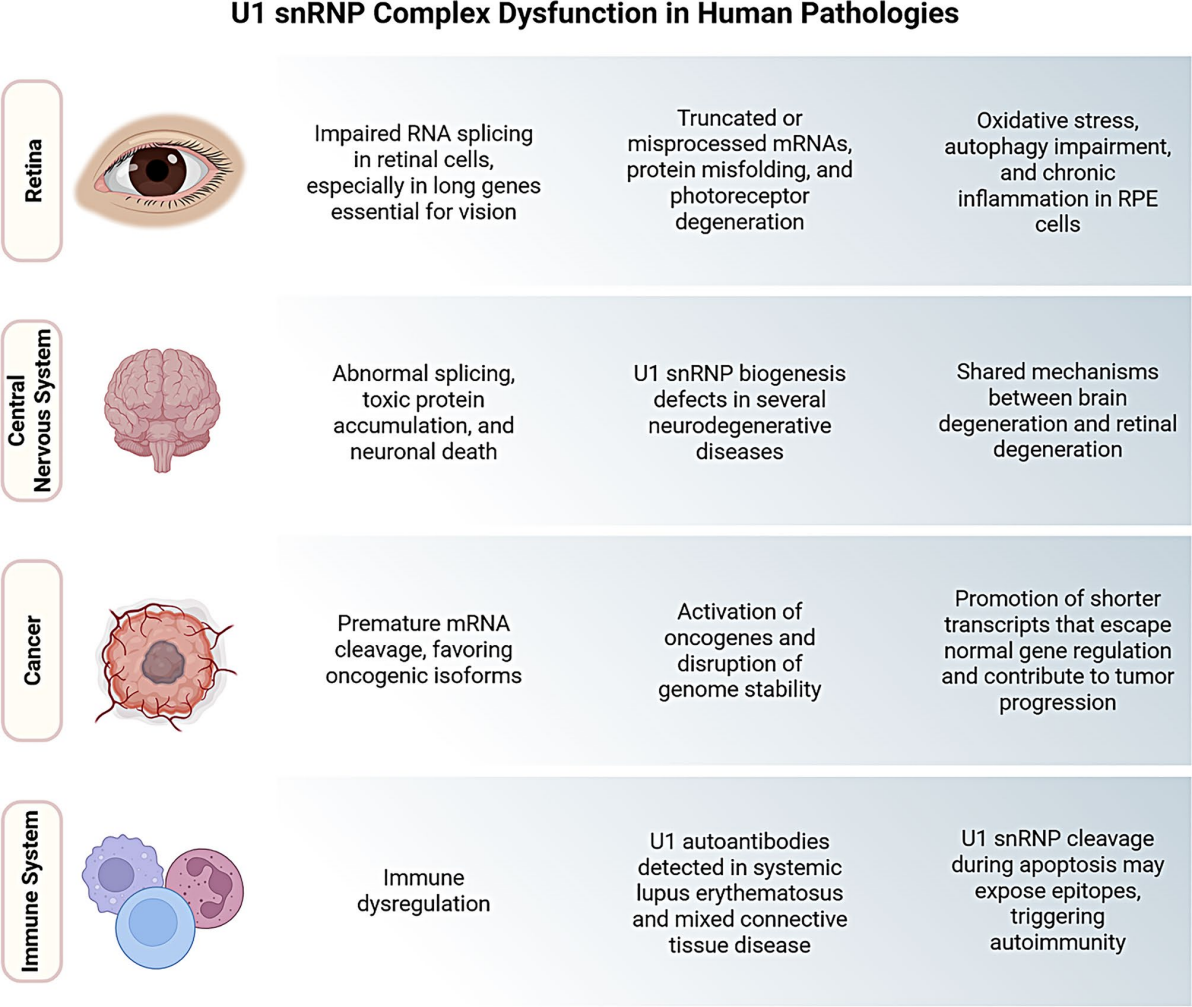
U1 snRNP complex dysfunction impairs RNA splicing, triggering widespread effects that contribute to eye diseases, CNS disorders, cancer, and autoimmune disorders, revealing its central role in cellular integrity. *created with* BioRender.com

Splicing defects in long genes have been implicated in several neurodegenerative disorders, including AD, PD, Huntington’s disease, amyotrophic lateral sclerosis (ALS), and spinal muscular atrophy (SMA). Disruption of U1 snRNP biogenesis and function has been observed in conditions such as AD, FUS-linked ALS, SMA, and pontocerebellar hypoplasia [42, 96, 135–137]. In addition to neurodegeneration, U1 snRNP dysfunction is also implicated in other human pathologies such as autoimmune diseases, as systemic lupus erythematosus and mixed connective tissue disease, where autoantibodies target U1 snRNP components, contributing to disease pathogenesis [138, 139]. Additionally, alterations in U1 snRNP expression or function have been observed in various cancers, influencing oncogenic splicing programs and genome stability (Fig. 6) [140–143].

Given that U1 snRNP is universally expressed across all cell types, the higher vulnerability of neurons to its dysregulation raises the question of why other cell types remain relatively unaffected. Neuronal susceptibility may arise from their dependence on the accurate expression of long genes essential for synaptic function and integrity [24, 59, 95, 104, 144–146].

In AD and other neurodegenerative disorders, changes in the levels of U1 snRNPs, particularly U1-70K (one of the components of the U1 complex), are associated with the dysregulation of genes essential for neuronal maintenance and synaptic function [147]. Many of these critical genes, such as those that encode the amyloid-beta (Aβ) peptide and TAU protein, are classified as long genes and contain numerous introns, making them especially reliant on precise splicing [54, 59, 148]. This is the case of the amyloid precursor protein (APP), which encodes the Aβ peptide. Under physiological conditions, APP plays important roles in neurons, including neurogenesis, synaptic plasticity, neurite outgrowth, and neuroprotection [149]. However, the abnormal processing of APP generates Aβ peptide isoforms that are prone to misfolding and aggregation. These peptides have been identified in drusen deposits in the brain and retina associated with both AD and AMD, supporting a shared pathogenic mechanism involving long misfolded proteins and chronic inflammation [150–153].

The microtubule-associated protein TAU (MAPT) gene encodes the TAU protein, which is essential for microtubule stability and function. This long gene undergoes extensive AS, which increases its functional complexity and results in the generation of multiple transcript isoforms. Disruptions in MAPT are associated with tauopathies, including AD and frontotemporal dementia. Abnormal TAU phosphorylation (p-TAU) results in the formation of neurofibrillary tangles, a hallmark pathological feature of these neurodegenerative diseases [154, 155]. Additionally, p-TAU is related to the disruption of cytoskeletal integrity in both brain tauopathies and retinal ganglion cells (RGCs), the primary cells affected by aging glaucomatous optic neuropathy (GON) [155–159]. Given their long axons, RGCs are highly dependent on TAU to maintain microtubule stability and efficient axonal transport, making them particularly vulnerable to TAU-related dysfunction [159, 160]. Owing to the importance of TAU in these processes, RGCs are disproportionately affected by p-TAU pathology compared with other retinal cells [161, 162].

Disruption of U1 snRNP biogenesis, a mechanism implicated in AD, may also contribute to retinal neurodegenerative disorders such as AMD and GON. This shared disruption, associated with pathological hallmarks as extracellular Aβ plaque accumulation and intracellular neurofibrillary tangles [148, 158, 159, 163–165] raises the hypothesis that AD, AMD, and GON may represent distinct phenotypic outcomes of a common underlying mechanism centered on U1 snRNP dysfunction, modulated by cell-type specificity. Targeting U1 snRNP regulation could thus represent a unifying therapeutic strategy for age-related neurodegenerative diseases affecting both the brain and retina.

## Implications for novel RNA therapeutic strategies for AMD

The retina has historically been central to RNA therapy development. The first ASO, fomivirsen, was approved in 1998 for intravitreal treatment of CMV retinitis [166]. Later, the field advanced with voretigene neparvovec (Luxturna, Spark Therapeutics), the first FDA-approved gene therapy for inherited retinal dystrophy, in 2017 [167]. Pegaptanib—the first anti-VEGF agent approved for neovascular AMD—validated the aptamer class [168]; the C5 inhibitor avacincaptad pegol has shown efficacy in slowing geographic-atrophy (GA) lesion growth [9, 169]; and the C3 inhibitor pegcetacoplan likewise reduces GA progression, but the magnitude of benefit remains modest and functional gains are limited, underscoring the need for upstream RNA-targeted advances [5].

Yet these approaches generally act downstream on single pathways. By contrast, U1 snRNP–targeted strategies address an upstream defect in RNA homeostasis. U1 snRNP safeguards full-length transcription and isoform fidelity—processes vulnerable in aging tissues such as the retina [31, 59].

Several U1-centric modalities (Table 2) remain at the pre-clinical stage but have corrected pathogenic splicing in IRD models: mutation-adapted/engineered U1 and exon-specific U1 (ExSpeU1) rescued defects in RHO (autosomal-dominant RP) and RPGR (X-linked RP) in patient cells and reporter systems; combining engineered U1 with ASO improved correction of a BBS1 splice-site mutation causing Bardet–Biedl–related rod–cone dystrophy; and adeno-associated virus (AAV)-delivered engineered U1 restored Opa1 expression with short-term ocular safety in an Opa1-mutant mouse model of autosomal-dominant optic atrophy [186, 187, 187–190, 194, 195]. By stabilizing transcriptomes rather than neutralizing single effectors, U1-directed therapies could surpass current options in scope and durability.

**Table 2:** U1-based and engineered U1 snRNA therapeutics: preclinical evidence

Therapeutic Area	RNA therapeutic	Strategy	Disease	Gene	Reference	Year
Dermatology	ExSpeU1	Restores LEKTI (SPINK5) splicing	Netherton syndrome	SPINK5	[170]	2015
Hematology	ExSpeU1	ExSpeU1 splice correction	Hemophilia B	F9	[171]	2016
	mutation‑adapted U1	Suppressor U1 enhances correct splicing at mutant donor	Fanconi anemia	FANCC	[172]	2010
	engineered U1 snRNA	Rescues exon‑8 definition disrupted by atypical mutations	Fanconi anemia	FANCA	[173]	2014
Hepatology	adapted U1/ExSpeU1	Efficient in‑vitro rescue of splice‑site mutations	PFIC1/BRIC1 spectrum	ATP8B1	[174]	2015
	engineered U1 snRNA	Somatic c.1061C > A counteracts c.1062 + 5 G > A enabling U1 rescue	Tyrosinemia type I	FAH	[175]	2018
	engineered U1 snRNA	Compensatory U1 5‘ss	Tyrosinemia type I	FAH	[176]	2020
Metabolic	mutation‑adapted U1	Compensatory U1 5‘ss	Propionic Acidemia	PCCA	[177]	2011
	engineered U1 snRNA	Modified U1 binds downstream enhancer to restore exon inclusion	Phenylketonuria	PAH	[178]	2018
Neurology	engineered U1 snRNA	Corrects DDC (AADC) splicing	AADC deficiency	DDC	[179]	2016
	ExSpeU1 (AAV9)	ExSpeU1 splice correction	Familial Dysautonomia	ELP1 (IKBKAP)	[180]	2018
	ExSpeU1	CDKL5 splicing rescue	CDKL5 Deficiency Disorder	CDKL5	[181]	2019
	ExSpeU1 (AAV9)	ELP1 exon‑20 splicing correction	Familial Dysautonomia	ELP1 (IKBKAP)	[182]	2022
	U1‑based (APT20TTMG)	Binds U1 snRNP	Alzheimer’s disease (preclinical)	U1 snRNP target (global)	[147]	2024
Oncology	U1 adaptor oligonucleotides	U1i gene silencing targeting BCL2 and GRM1	Melanoma	BCL2; GRM1	[183]	2013
	U1 adaptor oligonucleotides	U1i gene silencing targeting KRAS and MYC	Pancreatic cancer	KRAS; MYC	[184]	2017
	engineered U1	Low U1 dependence at NF1 exon‑29 donor	Neurofibromatosis type 1	NF1	[185]	2009
Ophthalmology	engineered U1 snRNA	Compensatory U1 5‘ss	Autosomal dominant Retinitis Pigmentosa	RHO	[186]	2009
	engineered U1 snRNA	Compensatory U1 5‘ss	X‑linked Retinitis Pigmentosa	RPGR	[187]	2011
	Engineered U1 + ASO	U1+ASO combined	Bardet–Biedl syndrome	BBS1	[188]	2019
	U1_asRNA (chimeric antisense U1)	U1_asRNA exon skipping	Retinitis pigmentosa (RPGR E9a)	RPGR	[189]	2022
	engineered U1 snRNA	AAV‑delivered engineered U1 corrects Opa1 splice defect in vivo	Autosomal Dominant Optic Atrophy	OPA1	[190]	2023
	ExSpeU1 (AAV2 intravitreal)	ExSpeU1 splice correction	Familial Dysautonomia –optic neuropathy (TgFD9)	ELP1 (IKBKAP)	[191]	2025
Pulmonology	ExSpeU1	ExSpeU1 splice correction	Cystic Fibrosis	CFTR	[192]	2012
	ExSpeU1	Rescue of common exon‑skipping CFTR mutations	Cystic Fibrosis	CFTR	[193]	2020

Table 2 summarizes studies that engage the U1 snRNP either through engineered U1 snRNA (compensatory U1 and ExSpeU1) or U1-targeting approaches (U1_asRNA, U1 adaptors/U1i, U1-binding modulators). Inclusion was limited to modalities that directly leverage U1 snRNP for splice correction, exon skipping, poly(A) interference, or related U1-mediated mechanisms. CRISPR editing, standard ASOs without U1 engagement, and general RNAi agents were excluded. This concise table complements the main text’s focus on U1 snRNP dysfunction. When multiple reports exist for a given program, the earliest peer-reviewed study that provides the clearest description of the modality is listed. Chekuri et al., 2025 [191] is a preprint flagged in the Reference field and should be interpreted with caution until peer review. Data cutoff: August 2025. Abbreviations: ExSpeU1, exon-specific U1; U1i, U1 interference; AAV, adeno-associated virus, U1_asRNA, U1 antisense RNA

APT20TTMG has a strategic sequence, structure, and chemical modifications to bind to U1 snRNP and pre-mRNAs’ conserved regions, ensuring the correct assembly during the splicing initiation process of all transcripts, without silencing or inhibiting genes.

Cross-disease evidence reinforces this rationale. Leal et al. (2024) [147] demonstrated that therapeutic correction of U1 snRNP function with a novel platform (APT20TTMG) in Alzheimer’s disease models not only normalized global splicing patterns but also downregulated aberrant expression of long genes vulnerable to premature transcriptional termination. APT20TTMG binds conserved U1 snRNP and pre-mRNA binding sites to stabilize spliceosome assembly at initiation across transcripts, without directly silencing genes. This treatment reduced pathological TAU accumulation in neurons, decreased Aβ burden, and lowered insoluble p-TAU across multiple brain regions. These findings suggest that restoring U1 snRNP integrity can re-establish transcriptomic homeostasis and attenuate hallmark neurodegenerative processes [147].

Given the molecular parallels between AD and AMD—including TAU phosphorylation, Aβ deposition, and long-gene vulnerability—these findings provide preclinical support for U1-targeted therapies as a unifying strategy across neurodegenerative disorders such as AD—where U1 dysfunction is well established—to age-related retinal disease, including AMD.

Translation to AMD will require optimized macular delivery (e.g., AAV vectors or chemically stabilized oligonucleotides), rigorous off-target and splice-isoform profiling, and validated biomarkers to monitor therapeutic impact. Nevertheless, by acting at an upstream regulatory node, U1-based therapeutics represent a promising next generation of RNA medicines for dry AMD [31, 59, 140, 147].

## Conclusions

Studying DNA alone is insufficient to uncover the complete genetic basis of complex diseases, such as AMD. The RNA transcriptome represents a vast layer beyond the DNA sequence that is now widely acknowledged and can drive research linking genetic variation to cellular pathology [47]. Future research is essential to unravel this complex relationship between neuronal function and dysregulation of RNA metabolism.

This review highlights the emerging role of the age-sensitive dysfunction of the U1 snRNP complex in the pathophysiology of AMD, proposing a model in which aging and environmental stressors can disrupt cotranscriptional and splicing processes.

Perturbation at this node unifies premature termination of long genes, isoform imbalance, chronic inflammation, as well as impaired autophagy and proteostasis, offering a mechanistic bridge to broader neurodegenerative conditions. By framing RNA metabolism dysregulation as a central driver of the pathology, this perspective introduces a novel therapeutic approach that may extend beyond AMD. It also provides insights into a potentially shared pathogenic mechanism and could contribute to addressing significant unmet medical needs across multiple neurodegenerative conditions.

## Data Availability

Not applicable.

## Abbreviations

AD: Alzheimer’s disease
Aβ: Amyloid-beta
ALS: Amyotrophic lateral sclerosis
AMD: Age-related macular degeneration
APP: Amyloid precursor protein
AS: Alternative splicing
ASO: Antisense oligonucleotide
AAV: Adeno-associated virus
ceRNA: Competing endogenous RNA
circRNA: Circular RNA
DNA: Deoxyribonucleic acid
ERGS: Engineered RNA-guided strategy
ExSpeU1: Exon-specific U1
GA: Geographic atrophy
GON: Glaucomatous optic neuropathy
IRD: Inherited retinal dystrophies
lincRNA: Long intergenic noncoding RNA
m6A: N6-methyladenosine
MAPT: Microtubule-associated protein TAU
miRNA: MicroRNA
mRNA: Messenger RNA
NATs/OS: Natural antisense transcripts/Opposite strand RNA
ncRNA: Noncoding RNA
p-TAU: TAU phosphorylation
PA: Polyadenylation
PD: Parkinson's disease
piRNA: PIWI-interacting RNA
PolyA: Polyadenosine
PR: Photoreceptor
pre-mRNA: precursor messenger RNA
RGC: Retinal ganglion cell
RNA: RNA polymerase II
RNA Pol II: PIWI-interacting RNA
RNP: Ribonucleoprotein
ROS: Reactive oxygen species
RP: Retinitis pigmentosa
RPE: Retinal pigment epithelial
rRNA: Ribosomal RNA
SF: Splicing factors
siRNA: Small interfering RNA
shRNA: Short hairpin RNA
piRNA: PIWI-interacting RNA
SMA: Spinal muscular atrophy
snoRNA: Small nucleolar RNA
snRNA: PIWI-interacting RNA
piRNA: Small nuclear RNA
snRNP: PIWI-interacting RNA
piRNA: Small nuclear ribonucleoprotein
tRNA: Transfer RNA
UTR: Untranslated region
U1i: U1 interference
U1_asRNA: U1 antisense RNA
VEGF: Vascular endothelial growth factors
